# Supplementary material for: Associations between Multimorbidity Patterns and Subsequent Labor Market Marginalization among Refugees and Swedish-Born Young Adults—A Nationwide Registered-Based Cohort Study
Source: J Pers Med. 2021 Dec 5;11(12):1305. doi: 10.3390/jpm11121305 (PMC8705997; doi:10.3390/jpm11121305)
Supplement: Supplementary file 1 [file jpm-11-01305-s001.zip › jpm-1421322-supplementary.pdf]

Supplement Table S1: List of ICD10 diagnostic groups in the multimorbidity network

ICD10 diagnostic groups*	Diagnoses Name
A00-A09	Intestinal infectious disease
A30-A49	Other bacterial diseases
A50-A64	Infections with a predominantly sexual model of transmission
B00-B09	Viral infections characterized by skin and mucous membrane
B15-B19	Viral hepatitis
B25-B34	Other viral diseases
B35-B49	Mycoses
B85-B89	Pediculosis, acariasis and other infestations
B95-B98	Bacterial, viral and other infectious agents
C00-C97	Malignant neoplasms
D00-D09	In situ neoplasms
D10-D36	Benign neoplasms
D37-D48	Neoplasms of uncertain or unknown behavior
D50-D53	Nutritional anemias
D60-D64	Aplastic and other anemias
D65-D69	Coagulation defects, purpura and other hemorrhagic condition
E00-E07	Disorder of thyroid gland
E10-E14	Diabetes mellitus
E20-E35	Disorders of other endocrine glands
E65-E68	Obesity and other hyperalimentation
E70-E90	Metabolic disorders
F00-F09	Organic, including symptomatic, mental disorders
F10-F19	Mental and behavioral disorders due to psychoactive substance uses

F20-F29	Schizophrenia, schizotypal and delusional disorders
F30-F39	Mood [affective] disorders
F40-F48	Neurotic, stress-related and somatoform disorders
F50-F59	Behavioral syndromes associated with physiological disturbances and physical factors
F60-F69	Disorders of adult personality and behavior
F70-F79	Mental retardation
F80-F89	Disorders of psychological development
F90-F98	Behavioral and emotional disorders with onset usually occurring in childhood and adolescence
F99-F99	Unspecified mental disorder
G40-G47	Episodic and paroxysmal disorders
G50-G59	Nerve, nerve root and plexus disorders
G90-G99	Other disorders of the nervous system
H00-H06	Disorders of eyelid, lacrimal system and orbit
H10-H13	Disorders of conjunctiva
H15-H22	Disorders of sclera, cornea, iris and ciliary body
H30-H36	Disorders of choroid and retina
H49-H52	Disorders of ocular muscles, binocular movement, accommodation and refraction
H53-H54	Visual disturbances and blindness
H60-H62	Diseases of external ear
H65-H75	Diseases of middle ear and mastoid
H80-H83	Diseases of inner ear
H90-H95	Other disorders of ear
I10-I15	Hypertensive diseases
I30-I52	Other forms of heart disease
I80-I89	Diseases of veins, lymphatic vessels and lymph nodes, not elsewhere classified
J00-J06	Acute upper respiratory infections
J09-J18	Influenza and pneumonia

J20-J22	Other acute lower respiratory infections
J30-J39	Other disease of upper respiratory tract
J40-J47	Chronic lower respiratory disease
K00-K14	Diseases of oral cavity, salivary glands and jaws
K20-K31	Diseases of esophagus, stomach and duodenum
K35-K38	Diseases of appendix
K40-K46	Hernia
K50-K52	Noninfective enteritis and colitis
K55-K64	Other diseases of intestines
K80-K87	Disorders of gallbladder, biliary tract and pancreas
K90-K93	Other diseases of the digestive system
L00-L08	Infections of the skin and subcutaneous tissue
L20-L30	Dermatitis and eczema
L40-L45	Papulosquamous disorders
L50-L54	Urticaria and erythema
L60-L75	Disorders of skin appendages
L80-L99	Other disorders of the skin and subcutaneous tissue
M00-M25	Arthropathies
M30-M36	Systemic connective tissue disorders
M40-M54	Dorsopathies
M60-M79	Soft tissue disorders
M80-M94	Osteopathies and chondropathies
M95-M99	Other disorders of the musculoskeletal system and connective tissue
N00-N08	Glomerular diseases
N10-N16	Renal tubulo-interstitial diseases
N17-N19	Renal failure
N20-N23	Urolithiasis
N30-N39	Other diseases of urinary system

N40-N51	Diseases of male genital organs
N60-N64	Disorders of breast
N70-N77	Inflammatory diseases of female pelvic organs
N80-N98	Noninflammatory disorders of female genital tract
O00-O08	Pregnancy with abortive outcome
O10-O16	Oedema, proteinuria and hypertensive disorders in pregnancy, childbirth and the puerperium
O20-O29	Other maternal disorders predominantly related to pregnancy
O30-O48	Maternal care related to the fetus and amniotic cavity and possible delivery problems
O60-O75	Complications of labor and delivery
O80-O84	Delivery
O85-O92	Complications predominantly related to the puerperium
O94-O99	Other obstetric conditions, not elsewhere classified
S00-S09	Injuries to the head
S10-S19	Injuries to the neck
S20-S29	Injuries to the thorax
S30-S39	Injuries to the abdomen, lower back, lumbar spine and pelvis
S40-S49	Injuries to the shoulder and upper arm
S50-S59	Injuries to the elbow and forearm
S60-S69	Injuries to the wrist and hand
S70-S79	Injuries to the hip and thigh
S80-S89	Injuries to the knee and lower leg
S90-S99	Injuries to the ankle and foot
T00-T07	Injuries involving multiple body regions
T08-T14	Injuries to unspecified part of trunk, limb or body region
T15-T19	Effects of foreign body entering through natural orifice
T36-T50	Poisoning by drugs, medicaments and biological substances
T51-T65	Toxic effects of substances chiefly nonmedicinal as to source
T66-T78	Other and unspecified effects of external causes

T80-T88	Complications of surgical and medical care, not elsewhere classified
T90-T98	Sequelae of injuries, of poisoning and of other consequences of external causes
V01-X59	Accidents
X60-X84	Intentional self-harm
X85-Y09	Assault
Y10-Y34	Event of undetermined intent
Y40-Y84	Complications of medical and surgical care
Y85-Y89	Sequalae of external causes of morbidity and mortality

*ICD10 diagnostic group indicates the International Classification of Diseases Tenth Edition provided by WHO

Supplement Table S2: The diagnoses classes with the highest relative risk of disability pension among 249,245 young adults in Sweden.

**Diagnosis Name**	ICD10 diagnostic groups*	n refugees	n Swedish -born	n individuals	n disability pension in refugees	n disability pension in Swedish-born	Relative risk in disability pension *
**Disorders of conjunctiva**	H10-H13	263	1202	1465	<10	12	2.67
**Diabetes mellitus**	E10-E14	128	1636	1764	<10	32	2.40
**Viral infections characterized by skin and mucous membrane lesions**	B00-B09	136	751	887	<10	10	2.21
**Organic, including symptomatic, mental disorders**	F00-F09	13	92	105	<10	20	1.77
**Diseases of veins, lymphatic vessels and lymph nodes, not elsewhere classified**	I80-I89	301	1186	1487	13	29	1.77
**Influenza and pneumonia**	J09-J18	354	998	1352	13	21	1.75

**Infections with a predominantly sexual mode of transmission**	A50-A64	692	5030	5722	10	42	1.73
**Sequelae of injuries, of poisoning and of other consequences of external causes**	T90-T98	304	1353	1657	11	32	1.53
**Papulosquamous disorders**	L40-L45	138	826	964	<10	<10	1.50
**Mental retardation**	F70-F79	41	124	165	19	39	1.47
**Toxic effects of substances chiefly nonmedicinal as to source**	T51-T65	85	563	648	<10	18	1.47
**Disorders of psychological development**	F80-F89	42	634	676	16	165	1.46
**Injuries to the thorax**	S20-S29	338	1389	1727	11	31	1.46
**Disorders of choroid and retina**	H30-H36	79	300	379	<10	11	1.38
**Disorders of gallbladder, biliary tract and pancreas**	K80-K87	167	783	950	<10	21	1.34
**Diseases of male genital organs**	N40-N51	381	2251	2632	<10	31	1.33
**Osteopathies and chondropathies**	M80-M94	179	888	1067	<10	15	1.32
**Diseases of external ear**	H60-H62	189	994	1183	<10	20	1.31
**Effects of foreign body entering through natural orifice**	T15-T19	176	1387	1563	<10	12	1.31
**Other forms of heart disease**	I30-I52	170	1081	1251	<10	30	1.27

*****ICD10 diagnostic group indicates the International Classification of Diseases Tenth Edition provided by WHO
* Relative risk (RRs) in disability pension indicates the risk of obtaining disability pension in refugee youth, compared to the matched Swedish-born in each diagnostic group.

Supplement Table S3: The diagnoses class with the highest relative risk of unemployment among 249,245 young adults in Sweden.

Diagnosis Name	ICD10 diagnostic groups*	n refugees	n Swedish -born	n individuals	n unemployment in refugees	n unemployment in Swedish-born	Relative risk in unemployment*
**Glomerular diseases**	N00-N08	35	200	235	10	<10	8.16
**Hypertensive diseases**	I10-I15	39	221	260	12	10	6.80
**Sequelae of external causes of morbidity and mortality**	Y85-Y89	237	1139	1376	63	48	6.31
**Diseases of appendix**	K35-K38	241	1443	1684	52	53	5.87
**Nutritional anemias**	D50-D53	199	291	490	39	10	5.70
**Mycoses**	B35-B49	574	1953	2527	115	71	5.51
**Other forms of heart disease**	I30-I52	170	1081	1251	38	45	5.37
**Other viral diseases**	B25-B34	381	1521	1902	79	61	5.17
**Sequelae of injuries. of poisoning and of other consequences of external causes**	T90-T98	304	1353	1657	67	59	5.05
**Effects of foreign body entering through natural orifice**	T15-T19	176	1387	1563	40	63	5.00
**Diseases of inner ear**	H80-H83	79	369	448	17	16	4.96
**Nerve. nerve root and plexus disorders**	G50-G59	159	722	881	38	35	4.93

**Noninfective enteritis and colitis**	K50-K52	215	1603	1818	44	67	4.90
**Injuries to the hip and thigh**	S70-S79	149	803	952	38	42	4.88
**Other disease of upper respiratory tract**	J30-J39	1317	6261	7578	220	215	4.86
**Chronic lower respiratory disease**	J40-J47	274	2680	2954	63	127	4.85
**Influenza and pneumonia**	J09-J18	354	998	1352	70	41	4.81
**Disorder of thyroid gland**	E00-E07	223	809	1032	51	39	4.74
**Acute upper respiratory infections**	J00-J06	655	3325	3980	125	139	4.57
**In situ neoplasms**	D00-D09	20	411	431	<10	23	4.47

*ICD10 diagnostic group indicates the International Classification of Diseases Tenth Edition provided by WHO
*Relative risk in long term unemployment indicates the risk of obtaining long term unemployment in refugee youth, compared to the matched Swedish-born in each diagnostic group.
